# THE ROLES OF COLATEC SOCIAL DANCING IN HEALTHY AGING AMONG KOREAN OLDER ADULTS

**DOI:** 10.1093/geroni/igad104.3056

**Published:** 2023-12-21

**Authors:** Milae Lee, Xingxing Wu, Laura Payne

**Affiliations:** University of Illinois Urbana-Champaign, Champaign, Illinois, United States; University of Illinois Urbana-Champaign, Champaign, Illinois, United States; University of Illinois Urbana-Champaign, Champaign, Illinois, United States

## Abstract

Leisure activities can contribute to healthy aging (An et al., 2022). However, little research exists on leisure activities with soft stigma (Kraus, 2010). Yet, social dancing in Colatec (i.e., Korean daytime discos for older adults) is becoming more popular among older Koreans. Using qualitative methods, we examined how Colatec social dancing affects Korean older adults’ healthy aging. Thirteen participants aged 60 to 75 who regularly social dance at Colatecs voluntarily participated in an in-depth semi-structured interview that lasted between 45 to 75 minutes. The audiotaped interviews were transcribed verbatim, coded, and analyzed following interpretive phenomenological analysis (Smith & Osborn, 2003). Trustworthiness was assessed with member checking and researcher triangulation. Results revealed that although it is still culturally stigmatized, Colatec social dancing was essential for participants’ healthy aging. Participants perceived numerous benefits from social dancing that motivated their enduring engagement, despite the risks of the social stigma associated with Colatec dancing. Themes that emerged included: 1) Exercise from social dancing alleviated stress and depressive feelings associated with negative life events; 2) Colatec dancing boosted many participants’ self-esteem, enhancing their overall wellbeing; and 3) Meaningful social connections were developed among participants. Findings align with existing literature on how moderate physical activity contributes to older adults’ health (Bauman et al., 2016). Moreover, leisure serves as a social lubricant and enhances healthy aging (Mitas et al., 2011). Lastly, perceived by participants as a “playground”, Colatec affords older Koreans a space for engaging leisure activity which facilitated their healthy aging process (Heo et al., 2018).

